# How does dancing promote brain reconditioning in the elderly?

**DOI:** 10.3389/fnagi.2013.00004

**Published:** 2013-02-26

**Authors:** Philip P. Foster

**Affiliations:** ^1^The Brown Foundation, Department of NanoMedicine and Biomedical Engineering, Institute of Molecular Medicine for the Prevention of Human Diseases, The University of Texas Health Science Center at HoustonHouston, TX, USA; ^2^Division of Pulmonary, Sleep Medicine, and Critical Care, Department of Internal Medicine, The University of Texas Health Science Center at HoustonHouston, TX, USA

“Iron rusts from disuse; water loses its purity from stagnation and freezes by cold even so does inaction sap the vigor of the mind”

(Leonardo da Vinci, 1452–1519)

Cognition encompasses multiple dimensions which may be defined as “connectome” which aims to achieve a complete connection mapping of the brain (Kuljiš, 2010). Jan-Christoph Kattenstroth, Hubert R. Dinse and their team at the Ruhr-University of Bochum, Germany, have developed a weekly “social” exercise protocol in healthy elderly conceptualized as one-hour dance class with a group for a six-month period (60 min/1 time/wk). Following the six-month protocol, significant improvements of performance were observed in cognition/attention (memory, visuo-spatial ability, language, and attention), reaction times, sensory-motor performance, posture and lifestyle but none in maximal aerobic capacity (VO_2max_) nor in fluid intelligence. Therefore, a question arose: how does the brain recondition in the elderly? Several hints may be inferred from those findings. The level of physical exercise inherent to dancing may not have been enough to produce an increase in aerobic fitness (VO_2max_) albeit the exercise-induced elevation of heart rate, cardiac output, and perfusion may have been sufficient to produce changes in protein expression (brain-derived neurotrophic factor, BDNF; cytokines; insulin-like-growth factors, IGF-1 and IGF-2) impacting cognitive plasticity (Chen et al., 2011; Foster et al., 2011).

Besides, dancing as a motion in a three-dimensional space relies on the path-integration which maintains permanent visual tracking of the direction and distance from reference points (landmark) during 3-D navigation in the environment (Hafting et al., 2005) and hence requires the activation of hippocampal and entorhinal networks. Motion and 3-D navigation in the environment are closely related to spatial memory. Deterioration of spatial memory is an early warning of cognitive impairment and potential onset of Alzheimer's disease. Specific cells, hippocampal place cells, are underlying spatial recognition in response to spatial stimulations such as environmental landmarks and translational or directional movement inputs (O'Keefe and Burgess, 1996). In dancing with uncharacterized motion and pacing, a few hypotheses may be set forth. We may speculate that during slow motion, place cells would fire in response to a unique, specific position in the environment (O'Keefe and Burgess, 1996; Doeller et al., 2010). Alternatively, grid cells, within entorhinal networks may be firing in response to a dance-induced rapid motion in multiple locations of the environment geometrically defined (Hafting et al., 2005). This geometrical superimposition of rapid motions onto a map of the environment is reproducing a pattern of equilateral triangles “tiling” the space (Hafting et al., 2005). Responses of grid cells are modulated by direction and speed of motion (Doeller et al., 2010). Indeed, stimulation provided by external landmarks may be a major player to improving spatial memory. Persistence of firing after sensory input has ceased suggests that network mechanisms are underlying grid cells executive mapping (Hafting et al., 2005). In humans, functional MRI suggests that direction and running speed are modulating the path integration (Doeller et al., 2010). Although the map is tied to external visual landmarks, it remains without them once composed (Doeller et al., 2010). Therefore, dancing may provide a benefit by input-stimulation following a simple exploration in motion of the surrounding environment. Depending on the importance of the virtual navigation paradigm in the stimulation process, a virtual training might enhance hippocampal and entorhinal volume and improve spatial cognition performance. However, it is unclear what would be the respective role of sub-maximal exercise and visual environmental landmark-mapping in the overall improvement found by the authors in dance classes.

Dance and music, as a form of art, are also molding brain circuits and may enhance cognition as well as emotional and behavioral patterns (Preminger, 2012). The long-term effect of auditory, multilingual, verbal, or musical workouts on the brain has been investigated underlying structural and functional adaptations (Jancke, 2009; Oechslin et al., 2010). One specialization related to musical training is the increase of gray matter in the auditory cortex (Schneider et al., 2005; Preminger, 2012). Several authors have also suggested that improvisation involving novel and complex situations such as controls of bearings and directions solicits frontal lobes (Preminger, 2012). Improvisation has also been used as training and rehabilitation of prefrontal functions (Preminger, 2012). Indeed, dancing as a neurocognitive experience activates multiple cognitive functions such as perception, emotion, executive function (decision-making), memory, and motor skills. A large array of brain networks is thus being activated.

Yet, how the brain achieves this remarkable feat in the elderly remains a puzzle, and questions about the respective roles of simultaneous mental (virtual reality) and skeletal muscle exercises are raised. Video game designers and movie directors, experts in virtual reality, put the emphasis on such widespread brain activation (Hasson et al., 2004, 2009; Hasson and Malach, 2006; Preminger, 2012). Virtual reality requires only minimal motor execution and cardio-respiratory activation compared with actual sustained sub-maximal skeletal muscle exercise. Furthermore, in virtual reality, a full stimulation of entorhinal networks by actual motion-induced navigation in the environment may thus be lacking. Another study (Anderson-Hanley et al., 2012) has also brought about insights into the role of a virtual reality-enhanced sub-maximal skeletal muscle exercise training for 3 months (45 min/5 times/wk at 60% heart rate reserve). Indeed, a 3-D virtual navigation on a computer screen while exercising on a stationary bicycle provided greater cognitive (executive) benefits than stationary bicycle alone. Strikingly, in Kattenstroth's study, one session per week is sufficient to bring about significant benefit. Such studies (Anderson-Hanley et al., 2012; Kattenstroth et al., 2013) have started to provide us with further insights into the more detailed physiological mechanisms involved. These studies speak in favor of one conclusion that may be summarized in the single most important question about brain reconditioning: how might these findings be reconciled?
